# Heredity—Some Reflections on It

**Published:** 1891-08

**Authors:** J. S. Dorset

**Affiliations:** Bonham, Texes, Late Superintendent Texas State Lunatic Asylum


### SELECTIONS.

### Heredity—Some Reflection on Its.

BY J. S. DORSET, M.D., BONHAM, TEXES.

Late Superintendent Texas State Lunatic Asylum,

Heredity is that biological law by which all beings endowed with life tend to repeat themselves in their descendants. "Will grapes grow upon thistles, or figs upon thorns? Can the offspring of the low and vicious, ignorant man be but like the fruit of the tree which brought it forth? That there are exceptions there is no doubt, but it is not the rule, and it is as hard for one born and raised in the environments of low degradation, morals and vice to be a pure, moral, refined, scholarly gentleman as it is for a "camel to pass through the eye of a needle."

Yet there is a species of moral depravity found in the children of the most refined; even the children of the clergy depart from their early training of precept and example of their parents, and the number is great. If we will look back, perhaps no farther than the grandfather, the traits so much to be deprecated may be found. A man may inherit his ancestor's intellect, his honors, his estate; but he must also take with these his diseases, vices, and other moral and physical obliquities. "The parents have eaten sour grapes, and their children's teeth are set on edge." These are some of the laws of heredity which are as immutable and unchangeable as the laws of the Medes and the Persians.

Take the dipsomaniac. While the great desire for alcohol may not develop in his offspring you may find it developed in another direction, such as idiocy or insanity; and if, perhaps, the immediate child may not exhibit mental or physical obliquity, wait; it will crop out in the grandchildren. Dipsomania, which used to be termed as merely a physiological condition, is now recognized by psychologists as a loss of will power, a disease of the brain, inherited or acquired, and the writer asserts, without fear of successful contradiction, it is a disease hereditary as any other, or as any characteristic in feature, limb or trunk.

When a child equally resembles father and mother the case needs no explanation ; it is the realization of the ideal law as far as that is possible. When he resembles one parent to the exclusion of the other, this exclusion does not really take place ; that parent whose influence appears to be lost may reappear in the next generation, or later. A man may have latent inherent traits of character undeveloped ; may marry and have children, and yet not discover the hidden spark; the wind which blows the ashes from the hidden spark of years will come; if not in his generation, it will come, even if later.

Darwin remarks truly that these facts oblige us to admit that certain characters, aptitudes and instincts may remain in the latent state in individuals, and even in a series of individual, while yet we are unable to find any trace of their presence ; and on this hypothesis, the transmission of a characteristic from grandfather to grandchild, with the apparent omission in the immediate parent of the oposite sex, becomes very plain. Frederick William I—the father of Frederick the Great—who was noted for his love of colossal men, dealt with his regiment of giants, as stockbreeders deal with their cattle. He would not allow his guards to marry women of stature inferior to their own; and the writer respectfully recommends this rule of Frederick William to all who contemplate taking as serious a step as marrying. But while these guards may have been giants in stature, they may have been Lilliputians in brain. So it will be as well to look well to the mental condition when you select a mother or father for your children, Heredity is a law, a natural law, and we may as well expect to escape co-equal law of nature—death—and live forever, as to dodge the law of heredity. If a man lives he shall die; nothing that has ever been can cease to be. This it is which fixes us in the indestructible law of causes and effect, and by which our poor personality is connected with the ultimate origin of things through an infinite concatenation of necessities. Heredity is but one form of that ultimate law which by physicists is called the conservation of energy, and by metaphysicians, universal casualty. The subject presents a large field yet unexplored, and for the present I must yield it to others more fitted to cultivate it properly than is the writer.—*Virginia Med. Monthly.*

